# 1,4-Disubstituted Thiosemicarbazide Derivatives are Potent Inhibitors of *Toxoplasma gondii* Proliferation

**DOI:** 10.3390/molecules19079926

**Published:** 2014-07-09

**Authors:** Katarzyna Dzitko, Agata Paneth, Tomasz Plech, Jakub Pawełczyk, Paweł Stączek, Joanna Stefańska, Piotr Paneth

**Affiliations:** 1 Department of Immunoparasitology, University of Lodz, Banacha 12/16, 90-237 Lodz, Poland; 2 Department of Organic Chemistry, Medical University, Chodźki 4a, 20-093 Lublin, Poland; E-Mails: agata.siwek@umlub.pl (A.P.); tomasz.plech@umlub.pl (T.P.); 3 Institute of Applied Radiation Chemistry, Technical University of Lodz, Zeromskiego 116, 90-924 Lodz, Poland; E-Mail: piotr.paneth@p.lodz.pl; 4 Institute for Medical Biology of the Polish Academy of Sciences, Lodowa 106, 93-232 Łódź, Poland; E-Mail: jpawelczyk@cbm.pan.pl; 5 Department of Genetics of Microorganisms, University of Lodz, Banacha 12/16, 90-237 Lodz, Poland; E-Mail: pstaczek@biol.uni.lodz.pl; 6 Department of Pharmaceutical Microbiology, Medical University of Warsaw, Oczki 3, 02-007 Warszawa, Poland; E-Mail: jstefanska@wum.edu.pl

**Keywords:** thiosemicarbazide derivatives, anti-*Toxoplasma gondii* activity, antibacterial activity, bacterial topoisomerases, toxicity, docking studies, DFT calculations

## Abstract

A series of 4-arylthiosemicarbazides substituted at the N1 position with a 5-membered heteroaryl ring was synthesized and evaluated *in vitro* for *T. gondii* inhibition proliferation and host cell cytotoxicity. At non**-**toxic concentrations for the host cells all studied compounds displayed excellent anti-parasitic effects when compared to sulfadiazine, indicating a high selectivity of their anti-*T. gondii* activity. The differences in bioactivity investigated by DFT calculations suggest that the inhibitory activity of 4-aryl-thiosemicarbazides towards *T. gondii* proliferation is connected with the electronic structure of the molecule. Further, these compounds were tested as potential antibacterial agents. No growth-inhibiting effect on any of the test microorganisms was observed for all the compounds, even at high concentrations.

## 1. Introduction

*Toxoplasma gondii* (*T. gondii*) is a worldwide distributed protozoan responsible for toxoplasmosis, one of the most prevalent parasitic infections in humans [1,2]. Typically, *T. gondii* infection in immunocompetent hosts is minor, self-limiting, and the parasite becomes dormant. However, it can have serious effects on immunocompromised individuals, such as HIV-AIDS positive, cancer or organ transplant human patients [2,3]. Under such conditions, *T. gondii* can result in life-threatening toxoplasmosis with *Toxoplasma* encephalitis and other complications (i.a. necrotic lesions within the central nervous system or retinochoroiditis) [4,5]. Moreover, women infected with *T. gondii* for the first time during pregnancy will pass the parasite on to the fetus. The estimated incidence of congenital toxoplasmosis in Poland was compatible with incidences (1 to 10 per 10,000 live births) reported in other European countries, in the United States, or in Japan. Congenital *T. gondii* infection may result in serious neurological and ophthalmic damage to the fetus or even, especially in the first three months of the pregnancy, spontaneous abortion. Furthermore, reactivation of undiagnosed congenital toxoplasmosis can lead to ocular toxoplasmosis later in life, in many cases causing blindness [6,7,8].

In spite of the severe consequences of toxoplasmosis, the therapy for this disease has not changed in the last 20 years. The current treatment involves the use of synergistic combinations of pyrimethamine, which inhibits the enzymatic activity of dihydrofolate reductase, and sulfonamides such as trimethoprim-sulfamethoxazole or sulfadiazine, whose target is dihydropteroate synthetase, supplemented with folinic acid [9,10]. The efficacy of this regime is limited, requiring the administration of relatively large amounts of drugs. Side effects include hypersensitivity, haematological toxicity, teratogenicity, allergic reactions, bone marrow suppression, and the development of resistance [11,12,13,14,15]. Furthermore, this treatment is not effective in eliminating the parasite located in the central nervous system [16,17]. An alternative is pyrimethamine in conjunction with clindamycin, spiramycin or atovaquone, but these drugs each possess their own limitations [8,9,10,11,12,13,14,15,16,17,18,19,20]. Thus, limited efficacy and side effects of existing drugs together with severe damage caused by *T. gondii* infection clearly indicates the need for development of new non-toxic, well-tolerated, and more efficacious therapeutic agents for controlling and curing toxoplasmosis.

**Figure 1:**
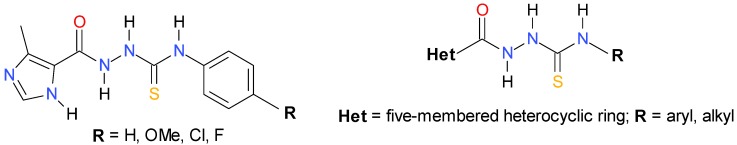
Left: Structures of 4-aryl-1-(4-methyl-1H-imidazole-5-yl)carbonylthiosemicarbazides with more potent anti-*Toxoplasma gondii* activity than the standard drugs sulfadiazine and hydroxyurea [21]; and right: Structures of title 4-aryl-1-hetarylcarbonylthiosemicarbazides.

Recently, Liesen *et al.* [21] have documented for the first time the anti-*T. gondii* activity of thiosemicarbazide-based compounds. According to the biological results, the tested compounds showed better LD_50_ values for both infected cells and intracellular parasites than the standard drugs sulfadiazine and hydroxyurea (see Figure 1). Since that time, no further reports have appeared in the literature describing the action thiosemicarbazides against *T. gondii*. Inspired by those results, efforts have been made by our research group to expand these initial findings with further details on anti-*T. gondii* activity of thiosemicarbazides. Herein, we present the outcomes of these investigations, as well as the results of subsequent DFT calculations, which allowed us to suggest that inhibitory activity of 4-arylthiosemicarbazides towards *T. gondii* proliferation is connected with the electronic structure of the molecule. In further studies, antibacterial activity of title thiosemicarbazide derivatives and inhibitory potency of one selected compound against bacterial type IIA topoisomerases are presented.

## 2. Results and Discussion

### 2.1. Chemistry

**Figure 2:**
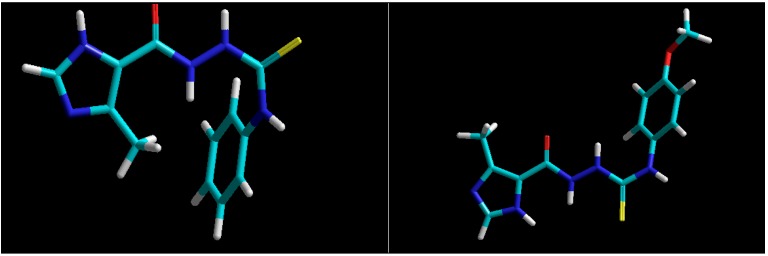
Structures of representative 4-aryl-1-(4-methyl-1H-imidazole-5-yl)carbonylthiosemicarbazides [21] with sulphur and oxygen atoms at C(=O)NHNHC(=S) core on the same side (**left**) or on the opposite side of the molecule (**right**).

As mentioned in the Introduction, recently Liesen *et al.* [21] documented for the first time the anti-*Toxoplasma gondii* activity of four 4-arylthiosemicarbazides with an imidazole ring at the N1 position (Figure 1). All studied compounds had a range of LD_50_ values between 0.05–5 mM for infected cells and 0.05–1.5 mM for parasites, indicating a more effective action than the standard drugs sulfadiazine (LC_50_ > 10 mM for infected cells, LC_50_ = 0.5 mM for intracellular parasites) and hydroxyurea (LC_50_ > 10 mM for infected cells, LC_50_ = 6 mM for intracellular parasites). According to the SAR analysis results, the electronic nature of the substituents on the phenyl ring of the 4-aryl-1-(4-methyl-1H-imidazole-5-yl)carbonylthiosemicarbazides seemed to have a low influence on bioactivity, thus indicating the dominant role of the imidazole moiety for modulating the bioactivity of the studied compounds. The promising bioassay results inspired us to design a series of 4-arylthiosemicarbazides with thiadiazole, thiophene or furan rings at the N1 position. Based on the results presented by Liesen *et al.* [21] we expected that the replacement of the imidazole core in 4-aryl-1-(4-methyl-1H-imidazole-5-yl)carbonylthiosemicarbazide with a similarly sized five-membered heteroaryl ring would result in compounds with a comparable bioactivity profile. The designed thiosemicarbazides presented structures similar to those proposed by Liesen *et al.* [21], with sulphur and oxygen atoms at C(=O)NHNHC(=S) core on the same side (Figure 2, left) or on the opposite side of the molecule (Figure 2, right), as confirmed by Amber calculations [22].

**Scheme 1:**
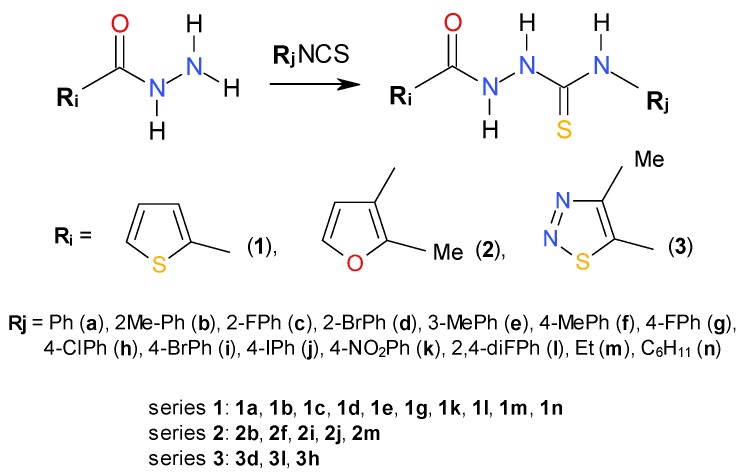
Synthetic route for 1,4-disubstituted thiosemicarbazides (series **1**–**3**).

Subsequently, these compounds were synthesized and Scheme 1 shows the synthetic route employed for their preparation. As can be seen, a simple synthesis was carried out, starting from the commercially available carboxylic acid hydrazides and the appropriate isothiocyanates, in ethanolic medium. At the end of the reaction, the isolated thiosemicarbazide derivatives were obtained as colourless solids. This procedure was adapted from an article previously reported by Plech *et al.* [23] and gave us satisfactory yields.

### 2.2. In Vitro Anti-Toxoplasma gondii Activity of the Title Compounds

In the next step of our studies, the prepared 1,4-disubstituted thiosemicarbazides (series **1**–**3**) were used to assess the inhibition of *T. gondii* growth *in vitro*. For this purpose, intracellular parasites (tachyzoites) of RH strain were incubated with different concentrations of the thiosemicarbazides ranging from 1 to 100 µg/mL. Inhibition of the parasite growth was monitored by measuring the specific incorporation of [^3^H]uracil in the parasite’s nucleic acids. Unfortunately, among the 15 compounds tested, derivatives **1a**–**1e**, **1k**, **1l**, **2f**, **2i**, **2j**, and **3h** were insoluble under the protocol conditions and, consequently, they were eliminated from further experiments. The percentages of the parasite growth inhibition in L929 cells by the remaining compounds **1g**, **2b**, **3d**, **3l** and the control drug-sulfadiazine as well as IC_50_ values are shown in Table 1.

**Table 1:** Effect of studied compounds on the intensity of *T. gondii* proliferation (%) in the L929 host cells.

Cmpd. No.	Assay: A. [^3^H] Uracil Incorporation B. qRT-PCR	Concentration [µg/mL]					IC_50_ [µg/mL]
		100	50	10	5	1	
**1g**	A.	nt	12.62 ± 4.78 *	44.71 ± 16.60 *	74.94 ± 7.00 *	96.14 ± 14.54	**33.17**
	B.	nt	5.00 ± 2.56 *	61.01 ± 11.23 *	nt	nt	nt
**2b**	A.	8.10 ± 2.78 *	58.78 ± 11.49 *	112.25 ± 22.91 *	107.95 ± 24.88	98.24 ± 23.83	**59.00**
	B.	7.29 ± 1.56 *	41.37 ± 9.98 *	nt	nt	nt	nt
**3d**	A.	36.52 ± 5.16 *	63.82 ± 5.32 *	83.84 ± 19.01	84.64 ± 15.68	90.03 ± 14.61	**74.93**
	B.	25.56 ± 6.32 *	75.13 ± 10.10 *	nt	nt	nt	nt
**3l**	A.	37.20 ± 7.47 *	88.34 ± 14.40 *	103.21 ± 19.27	85.76 ± 19.71	84.59 ± 16.61	**92.28**
	B.	14.08 ± 3.35 *	89.32 ± 15.74 *	nt	nt	nt	nt
**sulfadiazine**	A.	71.53 ± 8,94 *	78.73 ± 8,29 *	82.14 ± 11,26	79.18 ± 6,29 *	90.37 ± 11,65 *	>500 **
	B.	63.99 ± 10.58 *	nt	nt	nt	nt	nt
**2m**	A.	57.59 ± 15.02 *	66.49 ± 11.38 *	60.61 ± 9.56 *	75.74 ± 14.62	98.00 ± 19.53	**191.04**
	B.	53.03 ± 9.87 *	51.03 ± 7.02 *	nt	nt	nt	nt

nt: Not tested, * *p* < 0.05; to calculate the intensity of *T. gondii* proliferation compared to the untreated blank, the Equation was used: proliferation (%) = [100 × sample OD_570_ (the mean value of the measured optical density of the 1–100 µg/mL compounds of the test samples / blank OD_570_. (the mean value of the measured optical density of the untreated cells)]. IC_50_ [µg/mL]: Represents the concentration of tested compounds that was required for 50% of *T. gondii* proliferation inhibition *in vitro*. ** Numerous studies have shown that the drug susceptibility for parasites depended on the host cells used. Using as a model, RH strain and human MRC-5 cells [24,25], Vero [26] and HFF [27] as a host normal cells, the IC_50_ value of sulfadizaine reached from 2.5 to 77 μg/mL compared to human carcinoma HEp-2 and HeLa cells showing IC_50_ values between 600–700 μg/mL and >1000 μg/mL, respectively [28,29].

According to these results, all thiosemicarbazides **1g**, **2b**, **3d**, **3l** showed significant and reproducible anti-parasitic effects, with the assigned IC_50_ values 5- to 15-fold lower than those observed for sulfadiazine (IC_50_ > 500 µg/mL). Among them, a derivative with the thiophene ring of **1g** was found to be the most potent anti-*T. gondii* agent, with an IC_50_ of 33.17 µg/mL. When the thiophene scaffold was replaced with a similar in size furan moiety, as exemplified by **2b**, or a thiadiazole core, as exemplified by **3d**, **3l**, bioactivity was substantially lost. IC_50_ values determined for **2b**, **3d**, **3l**, however, were still at least 5-fold lower than the values obtained for sulfadiazine (see A in Table 1). High anti-parasitic activity of all selected thiosemicarbazides **1g**, **2b**, **3d**, and **3l** was also confirmed using a quantitative real-time PCR (qRT-PCR) assay (B in Table 1).

**Figure 3:**
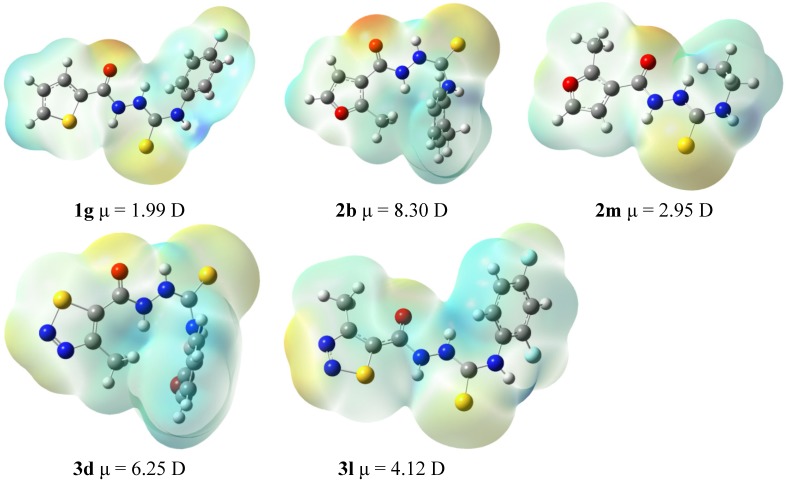
The electrostatic potential surfaces and dipole moments of **1g**, **2b**, **2m**, **3d**, and **3l**.

A preliminary assumption was thus put forward that the presence of both the five-membered hetaryl moiety at N1 position and the aryl moiety at N4 position of thiosemicarbazide core are the key functionalities required for potent anti-*T. gondii* activity of thiosemicarbazide-based compounds, which may be directly linked with the electronic structure of the molecule. In order to verify the preliminary hypothesis, 4-ethyl-1-(thiophen-2-yl)carbonylthiosemicarbazide (**1m**), 4-cyclohexyl-1-(thiophen-2-yl)carbonylthiosemicarbazide (**1n**), and 4-ethyl-1-(2-methylfuran-3-yl)carbonylthio-semicarbazide (**2m**) were synthesized and their bioactivity was tested. Unfortunately, derivatives **1m** and **1n** were insoluble under the protocol conditions and, consequently, they were eliminated from further experiments. Indeed, the replacement of the *ortho*-tolyl ring in **2b** with the ethyl chain as in **2m** resulted in a significant loss of bioactivity (IC_50_ 59.00 *vs.* 191.04 µg/mL, see Table 1 last entry), thereby confirming the validity of the aforementioned assumption. The assumption of the existence of the relationship between the anti-*T. gondii* activity of the studied thiosemicarbazides and their molecular structure was also confirmed based on DFT calculations. Figure 3 illustrates the electrostatic potential mapped on the density surface using the Gaussview program. Neutral fragments are characterized by the green color of the surface. The negative charge is represented by red, while the positive charge is marked in blue. If compound **1g** is excluded from the consideration, the biological activity correlates well with the dipole moment of the molecule. In this series it seems that the activity depends on the bimodal partial negative charge developed on the carbonyl oxygen and another moiety on the same side of the molecule (a sulfur atom in **2b** and **3d**). Its low value in **3l** and lack in **2m** may be responsible for the lower activity. However, the most active compound **1g** is characterized by the lowest dipole moment. It contains, however, two partial negative charge centers as the series discussed above and an additional such center on the fluorine atom. Comparison of the biological activity of **1g** and **2m** seems to indicate the crucial role of the second negative charge density on the top of the molecule on a moiety other than the carbonyl oxygen. Another observation that comes from the analysis of the presented structures is that the biological activity is pronounced when the methyl group of the five-membered ring is directed away from the carbonyl group. Since the number of compounds studied here is limited, the above observations can be treated as initial suggestions only and will be used as a guide for synthesis of other compounds in further studies.

In order to understand the molecular mechanism by which thiosemicarbazides induce their inhibitory activity towards *T. gondii*, the docking studies were carried out. The knowledge of unique aspects of *T. gondii* biochemistry and physiology has led to the identification of seven enzymes, essential for the survival, growth, replication, or viability of the microorganism, as reasonable targets for anti-*Toxoplasma* agents. According to the enzymatic studies, purine nucleoside phosphorylase (PNP) [30], adenosine kinase [EC.2.7.1.20] [31], dihydrofolate reductase (DHFR) [32,33], calcium-dependent protein kinase-1 (TgCDPK1) [34,35,36,37], 1-deoxy-_D_-xylulose-5-phosphate reductoisomerase (DXR) [38,39,40], enoyl reductase (TgENR) [41] were considered as attractive targets for discovering selective inhibitors to combat infections caused by this protozoan. Inspired by these results, based on the structures deposited in the Protein Data Bank, we analyzed the binding affinity of the title thiosemicarbazides with the active sites of aforementioned enzymes, with the exception of the model of dihydrofolate reductase (DHFR) binding site (PDB ID 4EIL) that retained several pathologies. The docking simulations were performed using the FlexX docking module [42] of the LeadIT environment as implemented in the BioSolveITprogram [43]. The total docking and hide scores of the studied compounds within the active sites of target enzymes together with H-bonds and close hydrophobic interactions are displayed in Table S1 in Supplementary Material. Although no structure-activity relationships (SAR) trends were observed when the docking conformations, the scores, and the interactions between thiosemicarbazide and residues of the binding site were analyzed in detail, all compounds were recognized as potential inhibitors of studied enzymes. To these enzymes, ribonucleotide reductase should also be included since this enzyme was identified as molecular target for anti-*T. gondii* activity of thiosemicarbazones—compounds closely related to thiosemicarbazides [21]. Of course, these above-mentioned proteins are only a very small cross-section of the potential protein targets within *T. gondii.* Evidently, enzymatic studies are necessary to develop our knowledge of the molecular basis of thiosemicarbazide derivatives bioactivity.

### 2.3. Cytotoxicity of **1g**, **2b**, **2m**, **3d**, and **3l** against L929 Cells

Since all studied compounds appeared to be more potent than control drug sulfadiazine, the assayfor the protozoan parasite *T. gondii* has been expanded to include a preliminary study on their *in vitro* host cell toxicity effects using an MTT assay. From a biological point of view, it is important for the studied compounds to inhibit the parasite growth at a low concentration and at the same time show no or low toxicity effects on host cells. Otherwise, the activity might be just due to the general toxicity, which disqualifies the compound as a drug or lead molecule candidate. Fortunately, from a comparison of the cytotoxicity test (CC_30_, Table 2) results and anti-*T. gondii* activity tests (Table 1), it can be seen clearly that all tested compounds inhibited the parasite growth at non-cytotoxic concentrations in host cells. The morphology of normal cells and the morphology of cells cultured with tested compounds are presented in Figure S1 in Supplementary Material.

**Table 2:** The viability of L929 cells (*in % of viable L929 cells*) in the concentration range of **1g**, **2b**, **2m**, **3d**, and **3l** between 1 µg/mL and 500 µg/mL *±* SD.

Cmpd. No.	Concentration [µg/mL]						CC_30_ [µg/mL]
	500	100	50	10	5	1	
**1g**	39.76 ± 5.83 *	67.00 ± 11.38 *	79.05 ± 2.89 *	97.32 ± 4.98	99.51 ± 5.16	102.52 ± 2.69	210.04
**2b**	30.28 ± 0.86	78.22 ± 1.61 *	84.54 ± 1.43 *	91.93 ± 5.21 *	94.01 ± 1.78 *	95.27 ± 9.52	187.51
**3d**	41.45 ± 5.16 *	79.53 ± 5.82 *	90.88 ± 6.71 *	97.51 ± 5.98	99.14 ± 0.82	102.36 ± 3.89	242.56
**3l**	48.02 ± 10.12 *	89.55 ± 3.44 *	99.04 ± 10.02 *	95.89 ± 0.39 *	92.87 ± 0.23 *	96.54 ± 0.69	285.52
**2m**	80.40 ± 2.74 *	92.17 ± 2.31 *	90.49 ± 2.99 *	94.05 ± 5.13	94.44 ± 2.62	104.05 ± 0.12	>500

* *p* < 0.05; To calculate the reduction of viability compared to the untreated blank the Equation was used: viability (%) = 100 × sample OD_570_ (the mean value of the measured optical density of the 1–100 µg/mL compounds of the test samples/blank OD_570_ ( the mean value of the measured optical density of the untreated cells). CC_30_[µg/mL]—represents the concentration of tested compounds that was required for 30% proliferation inhibition *in vitro*. The effect of tested compounds on the cell line L929 (%) was measured using MTT assay according to the international standards: ISO 10993-5:2009(E).

### 2.4. Antibacterial Activity and Inhibitory Potency against Bacterial Type IIA Topoisomerases

**Figure 4:**
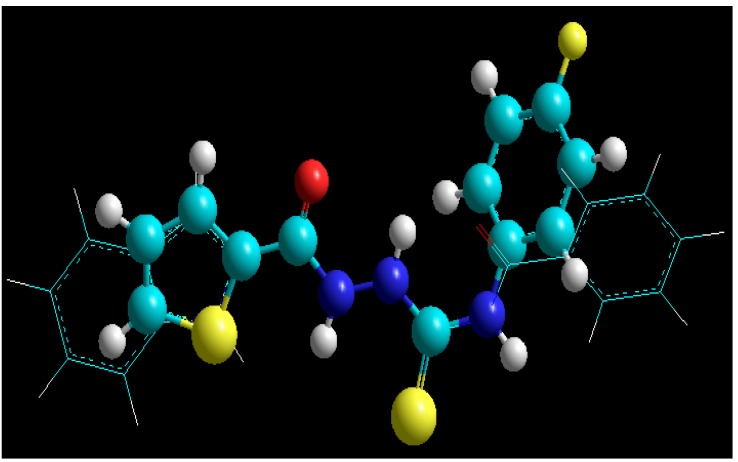
Overlay of the structures of known *S. aureus* topoisomerase IV inhibitor (*tubes*) [39] and 4-(4-fluorophenyl)-1-(thiophen-2-yl)carbonylthiosemicarbazide **1g** (*balls and sticks*).

In our resent papers [23,44] we have reported the antibacterial activity of thirty-one 4-arylthiosemicarbazides and found that some of tested compounds were effective against the reference strains of Gram-positive bacterial species and clinical isolates of *Staphylococcus aureus* with minimal inhibitory concentrations (MICs) in the range of 15.63–62.50 µg/mL. In addition, we have also reported two thiosemicarbazide derivatives [45], that is 4-benzoyl-1-(4-methyl-imidazol-5-yl)carbonylthiosemicarbazide with IC_50 _at 90 μg/mL and 4-benzoyl-1-(indol-2-yl)carbonyl-thiosemicarbazide with IC_50_ at 14 μg/mL, as initial prototypes of a novel class of inhibitors of bacterial topoisomerase IV—the enzyme that is essential for proper chromosome segregation and, consequently, to the survival of prokaryotic cells [46]. It was reasonable to suppose, therefore, that title compounds may also possess antibacterial activity. Thus, in addition to the antitoxoplasmic assays, we also tested antibacterial potency of **1g**, **2b**, and **2m** against panel of Gram-positive and Gram-negative bacterial strains. The antibacterial screening for compounds **3d** and **3l** was presented in our previous contribution [38] and it was found that both compounds were ineffective against all test microorganisms. Unfortunately, no growth-inhibiting effect on both Gram-positive and Gram negative bacterial strains was also observed for compounds **1g**, **2b**, and **2m** even at high concentrations. Nevertheless, as the geometry of molecule **1g** was similar to that of previously reported initial hits [39] (Figure 4), DNA gyrase and topoisomerase IV inhibition tests for **1g** were also conducted. No inhibitory effect, however, was observed against both bacterial enzymes.

## 3. Experimental

### 3.1. General Information

All commercial reactants and solvents were purchased from either Sigma-Aldrich ((St. Louis, MS, USA) or Lancaster ((Ward Hill, NY, USA) with the highest purity and used without further purification. Melting points were determined on a Fischer-Johns block and are uncorrected. Elemental analyses were performed by a AMZ-CHX elemental analyzer. IR spectra were recorded in KBr using a Specord IR-75 spectrophotometer. ^1^H-NMR spectra were recorded on a Bruker Avance (300 MHz). Analytical thin layer chromatography (TLC) was performed with Merc 60F_254_ silica gel plates and visualized by UV irradiation (254 nm).

### 3.2. Chemistry: General Procedure for Synthesis of 1-Substituted-4-arylthiosemicarbazides

A solution of 0.01 mol of carboxylic acid hydrazide and an equimolar amount of the appropriate isothiocyanate in anhydrous EtOH (25 mL) was heated under reflux for 30 min. Next, the solution was cooled and the solid formed was filtered off, washed with diethyl ether, dried, and crystallized from EtOH. Physicochemical characterizations of **1a**, **1c**, **1d**, **1g**, **1k**, **1l**, **1m**, **1n**, **2b**, **2f**, **2i**, **3d**, **3h**, and **3l** has been presented previously [38,47,48,49,50,51,52]. The structures of compounds **1b** (CAS number 891059-78-2) and **1e** (CAS number 903081-57-2) are known, however, there is no reference reporting their use or preparation or physico-chemical characterization, therefore their data have been included in this manuscript.

*4-(2-Methylphenyl)-1-(thiophen-2-yl)carbonylthiosemicarbazide* (**1b**). Yield: 94%. Mp: 155–157 °C. IR (ν, cm^−1^) 3354, 3337, 3308 (NH), 3084, 1601, 1586, 1527, 1485, 730 (Ar-H), 2922, 1349 (Aliph.), 1664 (C=O), 1245 (C=S), 775 (C–S). ^1^H-NMR (DMSO-d_6_) δ_H_ 2.19 (s, 3H, CH_3_), 7.10–7.23 (m, 5H, 4 × CH_ar_ & CH_thiophene_), 7.83–7.86 (m, 2H, 2 × CH_thiophene_), 9.62, 10.55 (2s, 3H, 3 × NH). Anal. Calcd for C_13_H_13_N_3_OS_2_ (291.39): C, 53.58; H, 4.50; N, 14.42. Found: C, 53.72; H, 4.59; N, 14.66.

*4-(3-Methylphenyl)-1-(thiophen-2-yl)carbonylthiosemicarbazide* (**1e**). Yield: 93%. Mp: 188–190 °C. IR (ν, cm^−1^) 3321, 3220 (NH), 3095, 3047, 1625, 1511, 725 (Ar-H), 2916, 1492, 1365 (Aliph.), 1664 (C=O), 1235 (C=S), 773 (C–S). ^1^H-NMR (DMSO-d_6_) δ_H_ 2.29 (s, 3H, CH_3_), 6.96-6.98 (m, 1H, CH_thiophene_), 7.17–7.29 (m, 4H, 2×CH_ar_), 7.84–7.87 (m, 2H, 2 × CH_thiophene_), 9.68, 9.81, 10.53 (2s, 3H, 3 × NH). Anal. Calcd for C_13_H_13_N_3_OS_2_ (291.39): C, 53.58; H, 4.50; N, 14.42. Found: C, 53.53; H, 4.66; N, 14.51.

### 3.3. Assay in Vitro for Anti-T. gondii Activity

#### 3.3.1. Animals

Inbred mice were kept under standard laboratory conventional conditions. All experimental procedures were conducted according to guidelines of the 9. Local Ethics Commission for Experiments on Animals in Lodz.

#### 3.3.2. Parasites

The tachyzoites of *T. gondii* strain RH – intraspecies type I (ATCC_ Number 50174™) were maintained through passages on female C57BL/6 (H-2^b^) and BALB/c (H-2^d^) mice with genetically determined high and low susceptibility (respectively) to *T. gondii* infection (10–12 weeks old). Tachyzoites washed out from the peritoneum cavity were once expanded *in vitro* on L929 cells.

#### 3.3.3. Influence of Thiosemicarbazide Derivatives on *T. gondii* Proliferation

L929, (2 × 10^4^ cells/100 µL/well) were grown in complete medium (IMDM) on 96-well plates. After 24 h incubation, a medium was removed and then *T*. *gondii* RH tachyzoites, suspended in culture medium supplemented with 1.0, 5.0, 10.0, 50.0 and 100.0 μg/mL 1,4-disubstituted thiosemicarbazides (**1g**, **2b**, **2m**, **3d**, **3l**) and as a control-sulfadiazine, were added (2 × 10^5^ tachyzoites/200 µL/well) to the cell monolayers. After subsequent 48 h incubation 1 µCi/well of [^3^H] uracil (Moravek Biochemicals Inc., Brea, CA, USA) was applied to each microculture for further 18–20 h. The amount of the isotope incorporated into the parasite nucleic acid pool, corresponding to the parasite growth, was measured by liquid scintillation counting with 1450 Microbeta Plus Liquid Scintillation Counter (Wallac Oy, Turku, Finland). The cpms of host cells alone (below 250/microculture) were subtracted from cpms of *T. gondii* infected microcultures.

### 3.4. Quantitative Real-Time PCR

#### 3.4.1. DNA Extraction

*T. gondii* genomic DNA was isolated from the tachyzoites of RH strain with Wizard^®^ SV Genomic DNA Purification System (Promega, Madison, WI, USA) according to the manufacturer’s instruction. The DNA concentration and purity were measured using a NanoPhotometer (Implen, München, Germany), while the integrity of the extracted DNA was tested using an ethidium bromide-stained agarose gel. DNA was used for the detection and quantitationof *T. gondii* in analyzed samples and it was stored at −20 °C until use.

#### 3.4.2. Detection in Infected Cells in the Presence of Tested Compounds

A quantitative real-time PCR (qRT-PCR) assay, targeting B1 gene [53] was performed to detect and to quantitate *T. gondii* in analyzed samples (**1g**, **2b**, **2m**, **3d**, **3l** and sulphadiazine) according to the modified protocol of Wahab *et al.* [54]. The forward primer GCATTGCCCGTCCAAACT, the reverse primer AGACTGTACGGAATGGAGACGAA and 5'-Fam-CAACAACTGCTCTAGCG-BHQ-1-3' probe were used. Amplification was carried out on a 7900HT real-time PCR system (Applied Biosystems, Carlsbad, CA, USA). The reaction mixtures (25 µL) consisted of 1x TaqMan PCR master mix supplemented with ROX (Applied Biosystems), 300 nM probe, 900 nM of each primer and 2 µL of template DNA. For quantification a standard curve was constructed using 10-fold serial dilutions of DNA extracted from a known number of *T. gondii*. The cycling parameters were: 50 °C for 2 min, initial activation at 95 °C for 10 min, and 45 two-step cycles of 95 °C for 15 s and 60 °C for 1 min. All samples were analyzed in triplicate.

### 3.5. Cytotoxic Assay

#### 3.5.1. Cell Culture

Cell line L929 (ATTC^®^ Catalog No. CCL-1, mouse fibroblasts) was routinely cultured in Iscove’s modified Dulbecco medium (IMDM, Cytogen, Princeton, NJ, USA), supplemented with 10% (v/v) fetal bovine serum (FBS, Sigma), plus 2 mM L-glutamine (Sigma), 100.0 U/mL penicillin (Sigma), 100.0 μg/mL streptomycin (Sigma), 5 × 10^−^^5^ M 2-mercaptoethanol (Sigma) and grown at 37 °C in a 10% CO_2_ humidified environment.

#### 3.5.2. Preparation of Compounds

Suspensions of the compounds **1g**, **2b**, **2m**, **3d**, **3l** and sulphadiazine were freshly prepared before the cells were exposed, and diluted 1–500 μg/mL with the culture medium (containing 2.5% DMSO). Cells treated with 2.5% DMSO-solvent served as a control in each experiment.

#### 3.5.3. Cell Viability Assay

The effects of tested compounds on the viability of mouse fibroblasts L929 cells were evaluated using the MTT [3-(4,5-dimethylthiazol-2-yl)-2,5-diphenyltetrazolium bromide] assay. The MTT assay was used according to international standards: ISO 10993-5:2009(E), *Biological evaluation of medical devices*, Part 5: *Tests for in vitro cytotoxicity*. L929 cells were placed into 96-well plates at a density of 1.0 × 10^4^/100 μL/well in culture medium and allowed to attach and form a confluent monolayer for 24 h before treatment. Afterwards, culture medium in the plates was replaced by 100 μL compounds suspension at concentration of 0–500 μg/mL and the cells were exposed for 24 h. Then, 1 mg/mL MTT (50 μL/well) was added to each well and incubated at 37 °C, 10% CO_2_ for 2 h. Mitochondrial dehydrogenases of viable cells reduced the yellowish water-soluble MTT to water-insoluble formazan crystals, which were solubilized with dimethyl sulfoxide (DMSO). The cell culture medium was aspirated cautiously, after which 150 μL DMSO was added to each well and mixed thoroughly. Optical density (OD) was read on the ELISA reader (Multiskan EX, Labsystems, Vienna, VA, USA) at 550 nm. The results were expressed as percentage viability compared with the treated 2.5% DMSO controls. All experiments were performed in triplicate.

### 3.6. Antibacterial Assay

The following microorganisms were used in this study: *Staphylococcus aureus* (ATCC 25923, ATCC 6538, ATCC 29213, NCTC 4163), *Staphylococcus epidermidis* ATCC 12228, *Bacillus subtilis* ATCC 6633, *Bacillus cereus* ATCC 11778, *Micrococcus luteus* (ATCC 9341, ATCC 10240), *Escherichia coli* (ATCC 10538, ATCC 25922, NCTC 8196), *Pemphigus vulgaris* NCTC 4635, *Pseudomonas aeruginosa* (ATCC 15442, ATCC 27853, NCTC 6749), and *Bordetella bronchiseptica* ATCC 4617. Initially, antibacterial activity of thiosemicarbazide derivatives was screened on the basis of growth inhibition zone (giz) utilizing the disc diffusion method, according to the Clinical and Laboratory Standards Institute guidelines [55]. For compounds showing the inhibitory effect on the growth of tested bacteria, monitored as an appearance of giz, the minimal inhibitory concentrations (MICs) were determined using agar dilution method, according to the Clinical and Laboratory Standards Institute guidelines [56]. The minimal inhibitory concentrations (MICs) were defined as the lowest concentration of the compound preventing growth of the tested microorganism. In both methods, recommended Mueller-Hinton II agar medium (Becton Dickinson, Heidelberg, Germany) was used. Solutions containing the tested agents were prepared in methanol or DMSO. Ciprofloxacin was used as control antimicrobial agent.

### 3.7. Inhibition of Bacterial Type IIA Topoisomerases

#### 3.7.1. Supercoiling Assays

The assays were performed using *S. aureus* Gyrase Supercoiling Assay Kits (Inspiralis, Norwich, UK). Briefly, supercoiled pBR322 plasmid DNA (0.5 mg) was incubated with 1 unit of gyrase, in the dedicated supercoiling assay buffer supplied by the manufacturer, in the presence of varying concentrations of the compounds tested. Reactions were carried out at 37 °C for 1 h and then terminated by the addition of equal volume of 2 × STOP Buffer (40% sucrose, 100 mM Tris-Cl pH 7.5, 1 mM EDTA, and 0.5 mg/mL bromophenol blue) and chloroform/isoamyl alcohol. Samples were vortexed, centrifuged and run through a 15 cm 1% agarose gel in TAE buffer (40 mM Trisacetate, 2 mM EDTA) for 3 h at 50 V. Gels were stained with ethidium bromide and visualized under UV light.

#### 3.7.2. Decatenation Assays

The assays were performed using *S. aureus* topoisomerase IV decatenation kits (Inspiralis). Interlinked kDNA substrate (0.5 mg) was incubated with 1 unit of topoisomerase IV (Inspiralis), in the dedicated decatenation assay buffer supplied by the manufacturer, in the presence of varying concentrations of the compounds tested. Reactions were carried out at 37 °C for 1 h and then terminated by the addition of equal volume of 2× STOP Buffer (40% sucrose, 100 mM Tris-Cl pH 7.5, 1 mM EDTA, 0.5 mg/mL bromophenol blue) and chloroform/isoamyl alcohol. Samples were vortexed, centrifuged and run through a 15 cm 1% agarose gel in TAE buffer for 1.5 h at 80 V. Gels were stained with ethidium bromide and visualized under UV light. The concentrations of the inhibitor that prevented 50% of the kinetoplast DNA from being converted into decatenated minicircles (IC_50_ values) were determined by plotting the results obtained from the densytometric analyses of the gel images using Quantity One software (BioRad, Hercules, CA, USA).

### 3.8. Data Analysis

The results of experiments (3.2. and 3.4.*)* were shown as a mean arithmetic values from 6–18 repeats (2–6 experiments) and were analyzed for statistical significance with the Statistica PL 5.0 software using the Mann-Whitney U test. During statistical verification, significance levels of * *p* < 0.05 were considered.

### 3.9. Computational Details

Conformational search was performed using the Amber force field as implemented in HyperChem 8.0.3. [57] and default convergence criteria. For the most stable conformers population analysis was carried out using the Merz-Kollman scheme [58] at the HF/6-31G theory level with the use of the Gaussian package [59].

### 3.10. Docking Studies

The docking simulations were performed using the FlexX docking module of the LeadIT environment as implemented in the BioSolveITprogram using models of following enzymes: purine nucleoside phosphorylase (PNP; PDB ID: 3MB8 ), adenosine kinase ([EC.2.7.1.20]; PDB ID: 1LII), calcium-dependent protein kinase-1 (TgCDPK1; PDB ID: 4M84), 1-deoxy-D-xylulose-5-phosphate reductoisomerase (DXR; PDB ID: 3AU9), enoyl reductase (TgENR; PDB ID: 2o2s), complexed with their reference ligands. The active sites were defined to include all atoms within 10 Å radius of the native ligands. To validate the docking protocol, ligands co-crystallized with the proteins were initially docked into the crystal structure of the appropriate enzymes; the best conformations obtained were practically identical with the experimental ones. Subsequently, studied compounds were docked using the same docking parameters. The first 100 top ranked docking poses were saved for each docking run.

## 4. Conclusions

In conclusion, a series of thiosemicarbazides was evaluated *in vitro* for inhibition of *T. gondii* proliferation and host cell cytotoxicity. All studied compounds displayed significant and reproducible anti-parasitic effects at concentrations that were non**-**toxic toward the host cells, with experimentally determined IC_50_ values at least twice (or even fifteen times) higher than that of sulfadiazine. DFT calculations seem to indicate that the activity depends on the bimodal partial negative charge developed on the carbonyl oxygen and another moiety on the same side of the molecule. Since the number of compounds studied here is limited, the above observations can be treated as initial suggestions and will be used as a guide for synthesis of other compounds in further studies.

## Supplementary Materials

Supplementary materials can be accessed at: http://www.mdpi.com/1420-3049/19/7/9926/s1.
