# Supplementary material for: 1,4-Disubstituted Thiosemicarbazide Derivatives are Potent Inhibitors of Toxoplasma gondii Proliferation
Source: Molecules. 2014 Jul 9;19(7):9926–43. doi: 10.3390/molecules19079926 (PMC6290556; doi:10.3390/molecules19079926)
Supplement: Supplementary file 1 [file molecules-19-09926-s002.pdf]

Supplementary Materials

Figure S1. Cytotoxic activity of 1,4-disubstituted thiosemicarbazide: **1g**, **2b**, **2m**, **3d**, and **3l**. Morphology of normal L929 cells and cultured cells with **1g** (50 µg/mL), **2b** (100 µg/mL), **2m** (100 µg/mL), **3d** (100 µg/mL), and **3l** (100 µg/mL).

control

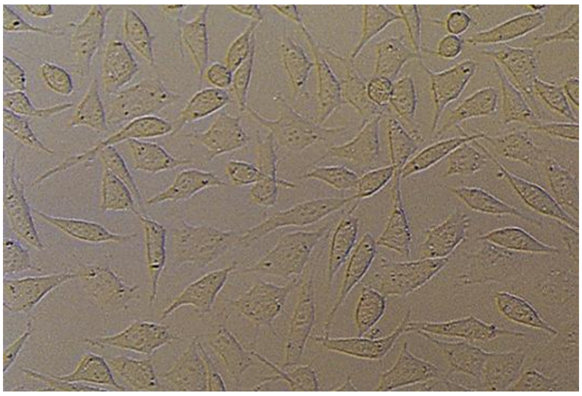

1g

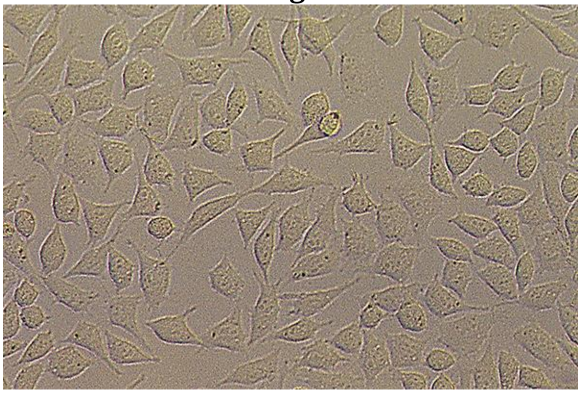

2b

2m

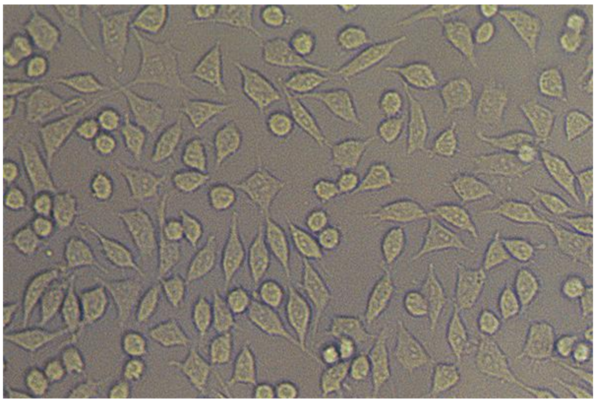

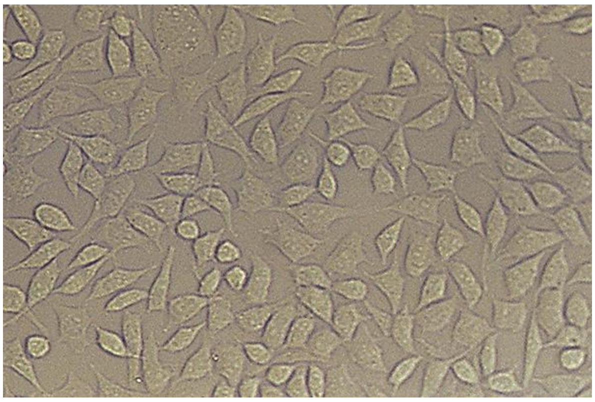

3d

3l

Table S1. The docking scores, free energies of binding and ligand efficiencies obtained from the Hyde scoring function, H-bond and close hydrophobic contacts corresponding to the best docking poses of compounds **1g**, **2b**, **2m**, **3d**, **3l** in the active site of purine nucleoside phosphorylase (PNP), adenosine kinase [EC.2.7.1.20], calcium-dependent protein kinase-1 (TgCDPK1), 1-deoxy-D-xylulose-5-phosphate reductoisomerase (DXR), and enoyl reductase (TgENR).

**Compound number**	**DS [*] ΔG [**] LE [***]**	**H-Bonds**			**Close van der Waals Contacts**
		**Compound**	**Residue**	**Distance (Å)**	
		**Purine Nucleoside Phosphorylase**			
**1g**	–25.02	C(=O)N$\underline{H}$NHC(=S)NH	H_2O	2.31	Thr96, Cys97,
	–13.00	C(=O)NHN$\underline{H}$C(=S)NH	Thr96	2.39	Trp216, Asp186,
	0.16	C(=O)NHNHC(=S)N$\underline{H}$	Thr96	2.06	Met187, Phe165,
					Gly98, Ile165
**2b**	–24.79	C(=O)NHN$\underline{H}$C(=S)NH	H_2O	1.58	Thr96, Cys97, Ile71,
	–24.00	C(=O)NHNHC(=S)N$\underline{H}$	Thr96	1.83	Asp186, Tyr166,
	0.28				Met187, Phe165

Table S1. *Cont.*

Compound number	DS * ΔG ** LE ***	H-Bonds			Close van der Waals Contacts
		Compound	Residue	Distance (Å)	
**2m**	–19.58	C(=O)N<u>H</u>NHC(=S)NH	H_2O	1.44	Ile185, Ile71,
	10.00	C(=O)NHN<u>H</u>C(=S)NH	Asp210	2.12	Asp186, Met187,
		C(=O)NHN<u>H</u>C(=S)NH	Asp210	2.00	Phe165, Trp216
	0.00	C(=O)NHNHC(=S)N<u>H</u>	Ile185	2.28	
**3d**	–26.21	C(=O)N<u>H</u>NHC(=S)NH	Asp210	2.12	Thr96, Cys97,
	–21.00	C(=O)NHN<u>H</u>C(=S)NH	$H_2PO_4^{2-}$	2.25	Met187, Phe165,
	0.25	C(=O)NHNHC(=S)N<u>H</u>	H_2O	1.64	Ile71
		<u>N2</u>-thiadiazole	H_2O	2.20	
**3l**	–25.76	C(=<u>O</u>)NHNHC(=S)NH	H_2O	1.85	Ala223, Cys97,
	–13.00	C(=O)NHN<u>H</u>C(=S)NH	H_2O	2.07	Asp186, Asp210,
	0.16	<u>N2</u>-thiadiazole	Lys232	2.17	Met187, Phe165,
		<u>N3</u>-thiadiazole	Lys232	2.13	Ile185, Thr96
		**adenosine kinase**			
**1g**	–30.47	C(=<u>O</u>)NHNHC(=S)NH	H_2O	2.28	Ala316, Gly280,
	–18.00	C(=<u>O</u>)NHNHC(=S)NH	H_2O	2.06	Val302, Val305,
	0.22	C(=<u>O</u>)NHNHC(=S)NH	Gly280	1.50	Ile349, His281,
		C(=O)NHN<u>H</u>C(=S)NH	Gly280	2.17	Thr278, Gly317,
		C(=O)NHNHC(=S)N<u>H</u>	Gly280	1.81	Asn223, Ile310
		S-thiophene	H_2O	2.27	
		S-thiophene	Asn223	2.35	
**2b**	–26.53	C(=<u>O</u>)NHNHC(=S)NH	H_2O	2.70	Gly315, Gly317,
	2.00	C(=<u>O</u>)NHNHC(=S)NH	H_2O	1.82	Mg999, Arg136,
	0.00	C(=<u>O</u>)NHNHC(=S)NH	Gly280	1.90	Thr278, Asn223,
		C(=O)NHN<u>H</u>C(=S)N<u>H</u>	H_2O	1.97	Ala316, Gly280
**2m**	–25.14	C(=<u>O</u>)NHNHC(=S)NH	H_2O	2.12	Gly317, Mg999,
	23.00	C(=O)NHN<u>H</u>C(=S)NH	H_2O	1.91	Ala316, Asn223
	0.00	O-furan	H_2O	1.93	
		O-furan	H_2O	3.73	
		O-furan	Gly280	2.15	
**3d**	–25.16	C(=O)N<u>H</u>NHC(=S)NH	H_2O	1.80	Val302, Thr278,
	–17.00	C(=O)NHN<u>H</u>C(=S)NH	Asn342	1.81	His281, Ile349,
	0.21	C(=O)NHNHC(=S)N<u>H</u>	Gly280	1.69	Gly280, Ala316
		N2-thiadiazole	H_2O	2.97	
		N2-thiadiazole	H_2O	2.18	
		N2-thiadiazole	Gly280	2.03	
		N3-thiadiazole	Thr278	3.04	
**3l**	–28.53	C(=O)N<u>H</u>NHC(=S)NH	H_2O	1.94	Val305, Val302,
	–16.00	C(=O)NHN<u>H</u>C(=S)NH	Asn342	2.10	Thr278, Ile349,
	0.18	C(=O)NHNHC(=S)N<u>H</u>	Gly280	1.99	Gly280, Ala345,
		N2-thiadiazole	H_2O	1.98	Gln346, His281,
		N2-thiadiazole	H_2O	3.03	Ile310
		N2-thiadiazole	Gly280	2.22	
		N3-thiadiazole	Thr278	1.92	

Table S1. *Cont.*

Compound number	DS * ΔG ** LE ***	H-Bonds			Close van der Waals Contacts
		Compound	Residue	Distance (Å)	
		**calcium-dependent protein kinase-1**			
**1g**	–23.98	C(=O)NHNHC(=S)NH	Glu178	2.34	Gly60, Gly58,
	–8.00	C(=O)NHNHC(=S)NH	Glu178	1.81	Val65, Lys59,
	0.10	C(=O)NHNHC(=S)NH	Lys59	1.78	Leu57, Gln341
		C(=O)NHNHC(=S)NH	Glu135	1.56	Glu178
		S-thiophene	Gln341	2.07	
**2b**	–23.65	C(=O)NHNHC(=S)NH	Gln341	2.24	Glu178, Gly60,
	–4.00	C(=O)NHNHC(=S)NH	Lys59	2.22	Lys59, Gln341,
	0.05	C(=O)NHNHC(=S)NH	Glu178	1.86	Gly58, Glu135,
		C(=O)NHNHC(=S)NH	Lys59	1.95	Ser61
		O-furane	Ser61	2.02	
**2m**	–25.14	C(=O)NHNHC(=S)NH	Lys59	1.98	Lys59, Gln341,
	23.00	C(=O)NHNHC(=S)NH	Glu135	1.89	Gly60, Ile194,
	0.00	C(=O)NHNHC(=S)NH	Lys59	1.86	Glu178
		O-furan	Ser61	2.10	
		O-furan	Lys80	2.51	
**3d**	–29.01	C(=O)NHNHC(=S)NH	Lys59	2.08	Gly60, Glu178,
	–5.00	C(=O)NHNHC(=S)NH	Glu135	1.58	Ile194, Lys59,
	0.06	C(=O)NHNHC(=S)NH	Glu178	1.52	Gln341
		N2-thiadiazole	Ser61	1.86	
		N2-thiadiazole	Lys80	2.35	
		N3-thiadiazole	Lys80	1.80	
**3l**	–31.05	C(=O)NHNHC(=S)NH	Lys59	2.08	Gly60, Ile194,
	20.00	C(=O)NHNHC(=S)NH	Glu135	1.59	Lys59, Glu178,
	0.00	C(=O)NHNHC(=S)NH	Glu178	1.57	Gln341
		N2-thiadiazole	Lys80	1.70	
		N3-thiadiazole	Lys80	2.34	
		N3-thiadiazole	Ser61	1.98	
		**1-deoxy-D-xylulose-5-phosphate reductoisomerase******			
**1g**	–21.82	C(=O)NHNHC(=S)NH	Lys297	2.21	Lys295, Trp296,
	12.00	C(=O)NHNHC(=S)NH	Pro358	1.90	Pro294, Lys297,
	0.00	C(=O)NHNHC(=S)NH	Lys295	1.66	Asn92, Asp359
		C(=O)NHNHC(=S)NH	Lys295	1.94	
		S-thiophene	Met360	2.51	
		S-thiophene	Asn92	2.33	
**2b**	–22.74	C(=O)NHNHC(=S)NH	Lys297	1.91	Asn92, Lys297,
	16.00	C(=O)NHNHC(=S)NH	Lys295	2.01	Pro358, Tyr357,
	0.00	C(=O)NHNHC(=S)NH	Pro358	1.87	Asp359
		O-furane	Asn92	1.81	
**2m**	–23.81	C(=O)NHNHC(=S)NH	Lys312	2.17	Trp296, Met298,
	4.80	C(=O)NHNHC(=S)NH	Asn311	2.50	Ndp501, Asn311,
	0.00	C(=O)NHNHC(=S)NH	Ser306	1.52	Ser270, Ser269,
		C(=O)NHNHC(=S)NH	Asn311	2.31	Ser306, Lys312
		C(=O)NHNHC(=S)NH	Met298	2.22	

Table S1. *Cont.*

Compound number	DS * ΔG ** LE ***	H-Bonds			Close van der Waals Contacts
		Compound	Residue	Distance (Å)	
**2m**	–23.81	C(=O)NHNHC(=S)NH	Lys312	2.17	Trp296, Met298,
	4.80	C(=O)NHNHC(=S)NH	Asn311	2.50	Ndp501, Asn311,
	0.00	C(=O)NHNHC(=S)NH	Ser306	1.52	Ser270, Ser269,
		C(=O)NHNHC(=S)NH	Asn311	2.31	Ser306, Lys312
		C(=O)NHNHC(=S)NH	Met298	2.22	
		O-furane	H_2O	1.61	
		O-furane	Ser270	1.54	
		O-furane	Ser269	1.77	
		O-furane	Ser270	2.12	
		O-furane	Gly271	2.20	
**3d**	–24.07	C(=O)NHNHC(=S)NH	Lys297	2.34	Trp296, Met298,
	1.00	C(=O)NHNHC(=S)NH	Pro358	1.75	Lys297, Trp296,
	0.00	C(=O)NHNHC(=S)NH	Lys295	1.39	Asp359, Tyr357,
		C(=O)NHNHC(=S)NH	Pro358	2.01	Pro358
		N2-thiadiazole	Asn92	1.86	
		N3-thiadiazole	Asn92	1.93	
**3l**	–19.73	C(=O)NHNHC(=S)NH	Met360	2.23	Met360, Asn92,
	60.00	C(=O)NHNHC(=S)NH	Pro358	1.75	Trp296, Lys297,
	0.00	C(=O)NHNHC(=S)NH	Lys295	1.53	Lys295
		N2-thiadiazole	H_2O	2.12	
		N3-thiadiazole	Asn92	2.27	
		**enoyl reductase**			
**1g**	–25.07	C(=O)NHNHC(=S)NH	Ala231	2.45	Ala231, Phe243,
	–5.00	C(=O)NHNHC(=S)NH	PO_4^-	2.32	Tyr179, Tyr189,
	0.07	S-thiophene	Gly131	2.21	Ile235, Val134,
		S-thiophene	Asn130	2.00	Ile244, Ala232,
					Nad500
**2b**	–22.23	C(=O)NHNHC(=S)NH	PO_4^-	1.80	Asn130, Nad500,
	–4.00	C(=O)NHNHC(=S)NH	PO_4^-	2.19	Val134, Tyr189,
	0.05	C(=O)NHNHC(=S)NH	Nad500	2.22	Met193, Ile235,
		C(=O)NHNHC(=S)NH	Ala129	2.11	Ala129, Ala232,
					Ala231
**2m**	–18.32	C(=O)NHNHC(=S)NH	Gly131	2.19	Asn130, Ala129,
	8.00	C(=O)NHNHC(=S)NH	Ala231	2.36	Ala231, Val134,
	0.00	C(=O)NHNHC(=S)NH	Ala129	1.88	Pro132, Gly131
**3d**	–25.52	C(=O)NHNHC(=S)NH	Tyr189	1.88	Asn130, Met193,
	–21	N2-thiadiazole	Asn130	1.85	Ala129, Ala232,
	0.25	N3-thiadiazole	Asn130	2.12	Phe243, Tyr189,
		N3-thiadiazole	Gly131	1.60	Ile235, Nad500
**3l**	–19.73	C(=O)NHNHC(=S)NH	Tyr189	2.24	Asn130, Met193,
	60.00	C(=O)NHNHC(=S)NH	Tyr189	2.50	Ala129, Ala232,
	0.00	N2-thiadiazole	Asn130	1.84	Phe243, Tyr189,
		N3-thiadiazole	Asn130	1.76	Ile235, Nad500,
		N3-thiadiazole	Gly131	2.27	Tyr179, Pro226,
					Ile244

* DS: docking score (in kcal/mol); ** ΔG (kJ/mol); *** LE: ligand efficiency; **** Plasmodium falciparum DXR enzyme, that was used for docking studies, shares an overall high degree of similarity with the *T. gondii* protein.
